# Inherent regional brain activity changes in male obstructive sleep apnea with mild cognitive impairment: A resting-state magnetic resonance study

**DOI:** 10.3389/fnagi.2022.1022628

**Published:** 2022-10-31

**Authors:** Yongqiang Shu, Xiang Liu, Pengfei Yu, Haijun Li, Wenfeng Duan, Zhipeng Wei, Kunyao Li, Wei Xie, Yaping Zeng, Dechang Peng

**Affiliations:** ^1^Department of Radiology, The First Affiliated Hospital of Nanchang University, Jiangxi, China; ^2^Big Data Center, The Second Affiliated Hospital of Nanchang University, Jiangxi, China; ^3^Department of PET Center, The First Affiliated Hospital of Nanchang University, Jiangxi, China

**Keywords:** obstructive sleep apnea, mild cognitive impairment, rs-fMRI, regional homogeneity, functional connectivity

## Abstract

Obstructive sleep apnea (OSA) is the most common sleep disorder worldwide. Previous studies have shown that OSA patients are often accompanied by cognitive function loss, and the underlying neurophysiological mechanism is still unclear. This study aimed to determine whether there are differences in regional homogeneity (Reho) and functional connectivity (FC) across the brain between OSA patients with MCI (OSA-MCI) and those without MCI (OSA-nMCI) and whether such differences can be used to distinguish the two groups. Resting state magnetic resonance data were collected from 48 OSA-MCI patients and 47 OSA-nMCI patients. The brain regions with significant differences in Reho and FC between the two groups were identified, and the Reho and FC features were combined with machine learning methods for classification. Compared with OSA-nMCI patients, OSA-MCI patients showed significantly lower Reho in bilateral lingual gyrus and left superior temporal gyrus. OSA-MCI patients also showed significantly lower FC between the bilateral lingual gyrus and bilateral cuneus, left superior temporal gyrus and left middle temporal gyrus, middle frontal gyrus, and bilateral posterior cingulate/calcarine/cerebellar anterior lobe. Based on Reho and FC features, logistic regression classification accuracy was 0.87; sensitivity, 0.70; specificity, 0.89; and area under the curve, 0.85. Correlation analysis showed that MoCA scale score in OSA patients was significant positive correlation sleep efficiency and negatively correlation with neck circumference. In conclusion, our results showed that the OSA-MCI group showed decreased Reho and FC in specific brain regions compared with the OSA-nMCI group, which may help to understand the underlying neuroimaging mechanism of OSA leading to cognitive dysfunction and may serve as a potential biomarker to distinguish whether OSA is accompanied by cognitive impairment.

## Introduction

Obstructive sleep apnea (OSA) is a highly common sleep disorder characterized by repeated partial collapse and obstruction of the upper respiratory tract, resulting in intermittent hypoxia, hypercapnia, and sleep fragmentation. OSA affects up to more than 30% of older adults (Peppard et al., 2013), with an incidence rate of ~14% in men and 5% in women (Heinzer et al., 2016). Long-term intermittent hypoxia is not only associated with various complications (hypertension, cardiovascular damage, and chronic kidney disease; Yayan et al., 2017), but can also lead to concomitant cognitive dysfunction, anxiety, and even Alzheimer’s disease (Daulatzai, 2015). Studies have also shown that OSA is associated with a variety of cognitive impairments, including deficits in attention, memory, executive function, visuospatial function, and language ability (Olaithe et al., 2018). However, the neuropathological mechanisms underlying cognitive dysfunction in OSA are not fully understood.

Neuroimaging studies to explain these cognitive deficits have revealed functional and structural changes in multiple brain regions in OSA patients (Kumar et al., 2012; Xia et al., 2016). Previous structural magnetic resonance imaging (sMRI) research has shown decreased white matter integrity and volume reduction (Lee et al., 2019) and regional cortical thinning in OSA patients (Joo et al., 2013). A resting-state function MRI (rs-fMRI) study to evaluate brain activity in the spontaneous state also showed that OSA patients had regional homogeneity changes (Santarnecchi et al., 2013) and abnormal global and regional functional connectivity (Park et al., 2016).It involves the default mode network (DMN), saliency network, and central executive network (Prilipko et al., 2011; Khazaie et al., 2017; Chen L. et al., 2018). Changes in the function and structure of these areas are associated with cognition, emotion, autonomic nerves, and sensory control (Chen L. T. et al., 2018; Yu et al., 2019; Kong et al., 2021). However, most previous studies have been limited to comparisons between OSA patients and healthy individuals, and functional differences between OSA patients with and without mild cognitive impairment (MCI) have not been assessed. Thus, how to account for the heterogeneity of concomitant cognitive impairment in the OSA population and the possible mechanisms linking OSA to MCI need to be clarified.

Rs-fMRI is a non-invasive technique that has become a hotspot in neuroimaging research owing to its high spatial and temporal resolution. Regional homogeneity (Reho) is a data-driven local measure of spontaneous neural activity that does not require the onset time of stimulation or prior knowledge and has good test–retest reliability (Xia et al., 2018). Functional connectivity (FC) is an fMRI data processing method used to explore the functional interactions between anatomically separated brain regions (Luo et al., 2021). Reho and FC are considered to be complementary in examining the synchronization of local and remote brain activity (Sun et al., 2018),and the simple computations and reliable representations involved have led to their use in exploring brain diseases with underlying functional alterations, such as epilepsy (Liu et al., 2021), Alzheimer’s (Ibrahim et al., 2021), and depression (Chen et al., 2021). Biomarkers obtained by various resting-state MRI techniques [functional connectivity, Reho, amplitude of low-frequency fluctuation (ALFF)] are widely used in disease classification (Hojjati et al., 2017, 2019; Ju et al., 2019; Wang et al., 2021). Long et al. used ALFF, regional uniformity, and gray matter density as classification features to improve the efficiency of MCI diagnosis by distinguishing between MCI patients and healthy controls. The classification model obtained high accuracy (sensitivity, 93.1%; specificity, 100%, area under the curve, 0.97; Long et al., 2016).

This study aimed to determine whether there are differences in regional consistency and FC across the brain between OSA patients with MCI (OSA-MCIs) and those without MCI (OSA-nMCIs) and whether such differences can be used to distinguish the two groups. We hypothesized that compare with OSA-nMCI, OSA-MCI would showed abnormal spontaneous functional activity and connectivity change. To test this hypothesis, the voxel-level Reho method was used to explore local spontaneous brain activity in OSA-MCI patients, while exploring functional connectivity with the whole brain using significantly different Reho brain regions as seed points. Then, logistic regression was used to evaluate whether Reho and FC values could be used as neuroimaging markers to distinguish whether MCI was associated with OSA.

## Materials and methods

As a descriptive cross-sectional study, we conducted an function analysis using Gpower software. The specific settings were: Effect size *d* = 0.8, *α* = 0.05, 1 − *β* = 0.8. The total sample size was required to be 52. In this study, we recruited a total of 95 OSA patients diagnosed in the sleep monitoring room of the Respiratory Department of the First Affiliated Hospital of Nanchang University between August 2017 and August 2022. Diagnosis was jointly determined by experienced respiratory physicians according to the American Society of Sleep Medicine 2017 Clinical Practice Guidelines for adult obstructive sleep apnea (Kapur et al., 2017). The inclusion criterion was male with apnea-hypopnea index (AHI) > 15/h. The exclusion criteria were as follows: (1) sleep disorders other than OSA (e.g., insomnia, somnolence, and circadian dysrhythmicity sleep disorder); (2) respiratory diseases, cardiovascular diseases, diabetes mellitus, hypothyroidism, central nervous system diseases, metabolic diseases, and tumors; (3) alcohol or illicit drug abuse or current use of psychotropic substances; (4) contraindications for MRI, such as claustrophobia and various internal stents; and (5) image artifacts (motion, metal). All participants were right-handed, native Chinese speakers, and aged 20–60 years.

This study was approved by the Medical Ethics Committee of the First Affiliated Hospital of Nanchang University [2020(94)] and was conducted according to the principles of the Declaration of Helsinki. Written informed consent was obtained from all participants.

### Polysomnography and neuropsychological assessment

All subjects were instructed to abstain from alcohol, coffee, and hypnotics prior to polysomnography monitoring. All participants underwent an overnight polysomnography (from 10 p.m. to 6 a.m. the next day) using a Respironics LE series physiological monitoring system (Alice 5 LE, Respironics, Orlando, FL, United States). Polysomnography monitoring included standard electrocardiogram, electroophthalmogram, electromyogram, electrocardiogram, oral and nasal airflow, chest and abdomen breathing movement, and snoring. Blood oxygen saturation (SaO2), sleep latency, total sleep time, sleep efficiency, sleep stage, arousal and respiratory events were also recorded.

Obstructive apnea was described as a sustained 90% reduction in airflow for ≥10 s with significant dyspnea. Hypopnea was defined as a ≥30% decrease in airflow for ≥10 s accompanied by an oxygen saturation of ≥4%. AHI was determined as the sum of apnea and hypopnea events per hour during sleep and was classified as mild (5–15), moderate (15–30), or severe (≥30; Berry et al., 2017). Cognitive function was assessed using the 11-item Montreal Cognitive Assessment (MoCA) scale (Chinese version) with the area under the ROC (AUC) of 0.930 (95%CI: 0.894; 0.965), a sensitivity of 92%, and a specificity of 85% (Hu et al., 2013). All MoCA scale assessments were performed by one senior experienced psychologists. Briefly, the MoCA scale examines eight cognitive domains, including executive functioning, language, attention, computation, abstraction, naming, memory, and orientation. A total MoCA score of <26 indicates cognitive impairment (Chen et al., 2016).

### MRI data acquistion

All patients underwent MRI using a 3.0 Tesla MRI scanner with an 8-channel phased array head coil (Siemens, Munich, Germany) at our hospital. Foam pads and earplugs were used to reduce head movement and noise. Before the scan, the patients were instructed to close their eyes, stay awake, and not engage in specific thinking. First, a routine MRI scan was performed. The conventional T1-weighted imaging parameters were as follows: repetition time (TR) = 250 ms, echo time (TE) = 2.46 ms, thickness = 5 mm, gap = 1.5 mm, field of view (FOV) = 230 × 230 mm. For T2-weighted imaging, they were: TR = 4,000 ms, TE = 113 ms, thickness = 5 mm, gap = 1.5 mm, FOV = 230 × 230 mm, slice = 19. Then, high-resolution T1-weighted MRI images of brain structures were obtained using sagittal brain volume sequences (TR = 1,900 ms, TE = 2.26 ms, thickness = 1.0 mm, gap = 0.5 mm, FOV = 250 × 250 mm, Matrix = 256 × 256, turning angle = 9°, slice = 176). Finally, the rs-fMRI data were collected in the echo plane imaging sequence (TR = 2,000 ms, TE = 30 ms, flip angle = 90°, thickness = 4.0 mm, gap = 1.2 mm, FOV = 230 × 230 mm2, matrix size = 64 × 64, slice = 30). A total of 240 rs-fMRI images were obtained. Two experienced radiologists read the images to exclude macroscopic lesions (demyelinating encephalopathy, brain tumors) and motion artifacts.

### Data preprocess

Imaging data were examined using MRIcro software^1^ to discard suboptimal data, such as the presence of deflected or missing images, etc. Data processing is based on Data Processing & Analysis for Brain Imaging 6.0 (DPABI6.0, Chinese Academy of Sciences, Beijing, China, http://rfmri.org/dpabi), which is based on statistical parameter mapping (SPM12, http://www.fil.ion.ucl.ac.uk/spm/software/spm12/), and in MATLAB2018b (Math Works, Natick, MA, United States). Firstly, the file format was converted from Digital Imaging and Communications in Medicine to Neuroimaging Informatics Technology Initiative. Secondly, The first 10 time points are removed to ensure signal stability. Then, time layer correction and 3D head motion correction were performed. Patients whose frame displacement exceeded 2.5 standard deviations were excluded (Van Dijk et al., 2012). Structural images were co-registered with functional images for each subject using a linear transformation. Thus, the new segmentation in SPM12 was used to segment the structural images of all subjects into grey matter, white matter and CSF. Then, the image space was normalized to the Montreal Neurological Institute (MNI) template and resampled to 3 × 3 × 3 mm voxels. Finally, the Friston 24 parameters, white matter signals, and cerebrospinal fluid signals were regressed from the time series of all voxels using linear regression, after filtering with a temporal filter (0.01–0.08 Hz). The data preprocessing is described in detail in our previous study (Li et al., 2021).

The Reho value was generated using Kenndall’s consistency coefficient to generate a separate Reho map (in a given voxel and its 26 nearest time series; Zang et al., 2004). The calculation formula is as follows:


$$\mathrm{ReHo}=\frac{\sum {\left(R_{i}\right)}^{2}-n{\left(\bar{R}\right)}^{2}}{K^{2}\left(n^{2}-n\right)/12}$$

where Reho is the consistency coefficient of Kenndall in a given voxel, and the range is 0–1; *K* is the voxel number of the time series in the measurement cluster, *k* = 27; n is the rank number; and Ri is the total rank of the ith time point, where $\bar{R}$ = (*n* + 1)*K*/2 is the average value of Ri. When a given cluster is consistent with its neighbors in the time series, the consistency coefficient is close to 1. Then, to reduce the global effect of subject variability, we divided the Reho plot by the Reho value of the global tie and spatially smoothed with a gaussian kernel (half-height full-width = 6 mm). Reho plots were generated using Fisher’s r-to-z normalization, and szReho plots were used for final statistical analyses.

### Functional connectivity analysis

Functional connectivity analysis was calculated using DPABI 6.0. The brain areas that exhibited significantly different Reho values between the OSA-MCI and OSA-nMCI groups were set as regions of interest (ROIs). First, the time series of each region (i.e., the average of the fMRI sequences of all voxels in that region) were extracted. Then, Pearson’s correlation coefficients between the time series and all other voxels in the brain were calculated to obtain a whole-brain FC map of the subject. Finally, Fish R-to-z changes were used to transform the correlation maps into Z-value maps.

### Logistic regression

Logistic regression (LR) is a multivariable technique that requires a functional relationship between a number of predictor variables and a single output to predict the likelihood of a target variable. Reho and FC values, which were significantly different between OSA-MCI and OSA-nMCI groups, were used as features to construct LR models. First, we calculated the Pearson correlation coefficient between the characteristics of each group, with a 0.75 cutoff for the related absolute threshold. Given that the characteristics of correlation is greater than the threshold after comparing the mean absolute correlation, the mean absolute correlation variable was deleted. To weaken the multicollinearity at the expense of the smaller variable, the most valuable predictor variable was retained. Mesh optimization was used to optimize the hyperparameters, and leave-one-out cross validation and permutation test (5,000 times) were used to verify the model performance. The accuracy, sensitivity and specificity of the model were calculated, and the predictive performance of the model was evaluated according to the receiver operating characteristic curve and the area under the curve. The heterogeneity of the test results was evaluated by Kappa.

### Statistics

For demographic and clinical evaluation data, we first processed the data using SPSS 23.0 software and tested the normality of the data by Kolmogorov–Smirnov. Then, two-sample *t*-test was performed on the data conforming to normal distribution, and Mann–Whitney *U* test was performed on the data not normally distributed. *p* < 0.05 was considered statistically significant.

Voxel-based comparisons of all Reho maps and functional connections were performed using DPABI6.0. Two-sample *t*-tests were performed to analyze differences in the Reho and FC maps among the groups. Multiple comparisons were corrected using the Gaussian random field method (voxel level, *p* < 0.005; cluster level, *p* < 0.05).

Furthermore, Pearson correlation analysis was performed to evaluate the relationship between clinical variables, MoCA scale scores and the abnormal signal value in Reho and FC with significant group differences. All statistical analyses were performed using SPSS 23.0 with a statistical significance level of *p* < 0.05, and the analyses were corrected for multiple comparisons using the Bonferroni correction.

## Results

### Demographic and clinical characteristics

There were significant differences in neck circumference (*p* = 0.018) and MoCA scale score (*p* < 0.001) between the OSA-MCI and OSA-nMCI groups. Meanwhile, there were no between-group significant differences in age, education, BMI, waist circumference, AHI, LSaO2, MSaO2, SaO2 < 90%, sleep efficiency, oxygen index reduction, and AI (all *p* > 0.05; Table 1).

**Table 1:** General clinical scale.

	OSA-MCI *n* = 48	OSA-nMCI *n* = 47	Value of *p*
Age^a^, years	38.37 ± 8.06	35.48 ± 8.86	0.100
education^b^	13.06 ± 2.31	13.59 ± 3.13	0.347
BMI^a^	27.35 ± 3.08	26.91 ± 4.13	0.561
Neck circumference^b^	41.47 ± 2.92	40.10 ± 2.60	0.018
Waistline^b^	99.66 ± 6.79	97.59 ± 18.78	0.383
AHI^a^	54.38 ± 23.26	50.29 ± 18.78	0.349
LSaO_2_^b^	70.77 ± 12.46	68.00 ± 12.38	0.280
MSaO_2_^b^	92.21 ± 3.58	91.71 ± 5.13	0.580
Sleep efficiency%^b^	78.91 ± 22.63	84.86 ± 12.04	0.114
AI^b^	30.52 ± 17.75	33.59 ± 21.78	0.453
Oxygen index reduction^b^	47.40 ± 23.09	43.34 ± 23.43	0.825
SaO_2_ < 90%^b^	24.94 ± 19.88	23.67 ± 16.25	0.728
MoCA^b^	22.4 ± 2.36	27.27 ± 1.17	<0.001

AHI, apnea hypopnea index; LSaO_2_, minimum blood oxygen saturation; MSaO_2_, average blood oxygen saturation; SaO_2_ < 90%, percentage of total sleep time with oxygen saturation <90.

a Student, *t*-test.

b Mann–Whitney *U*-test.

### Group differences in Reho maps

**Figure 1:**
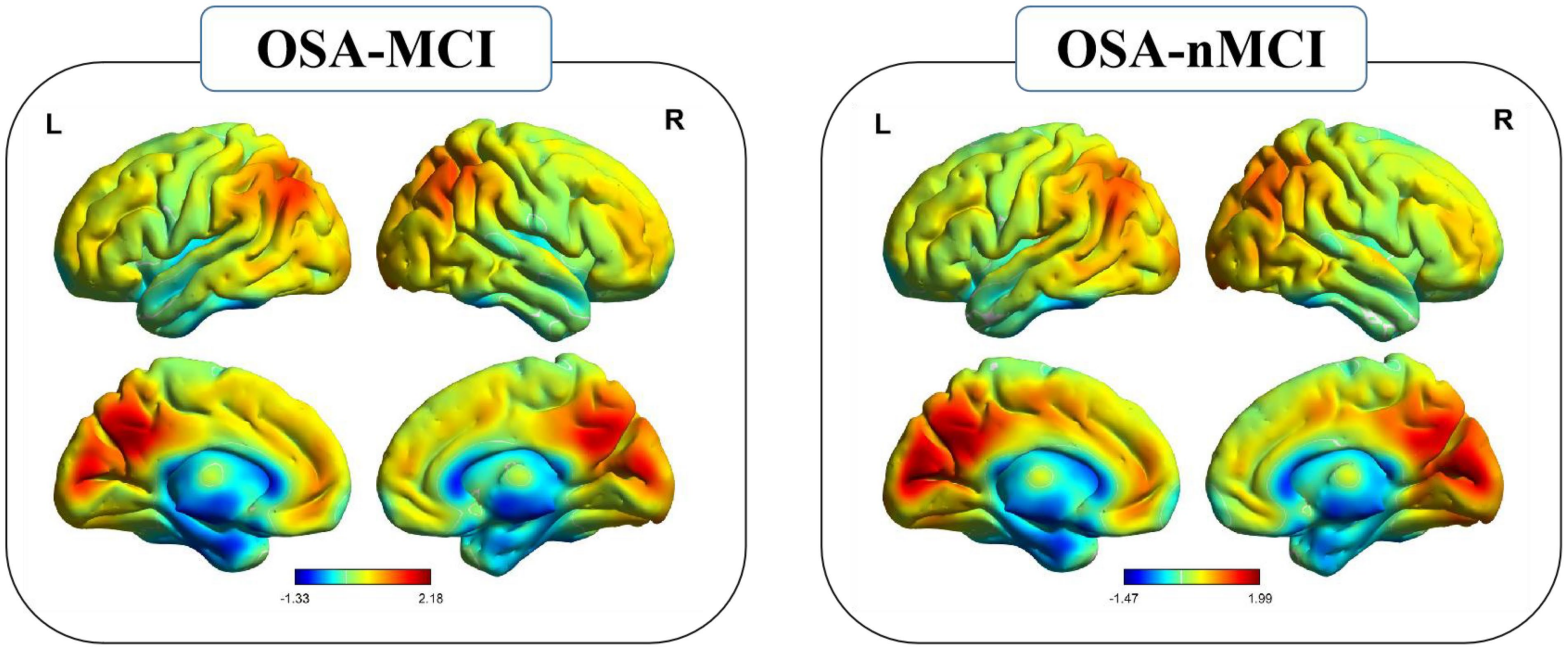
Reho spatial patterns at the group mean level of the OSA-MCI and OSA-nMCI groups.

**Figure 2:**
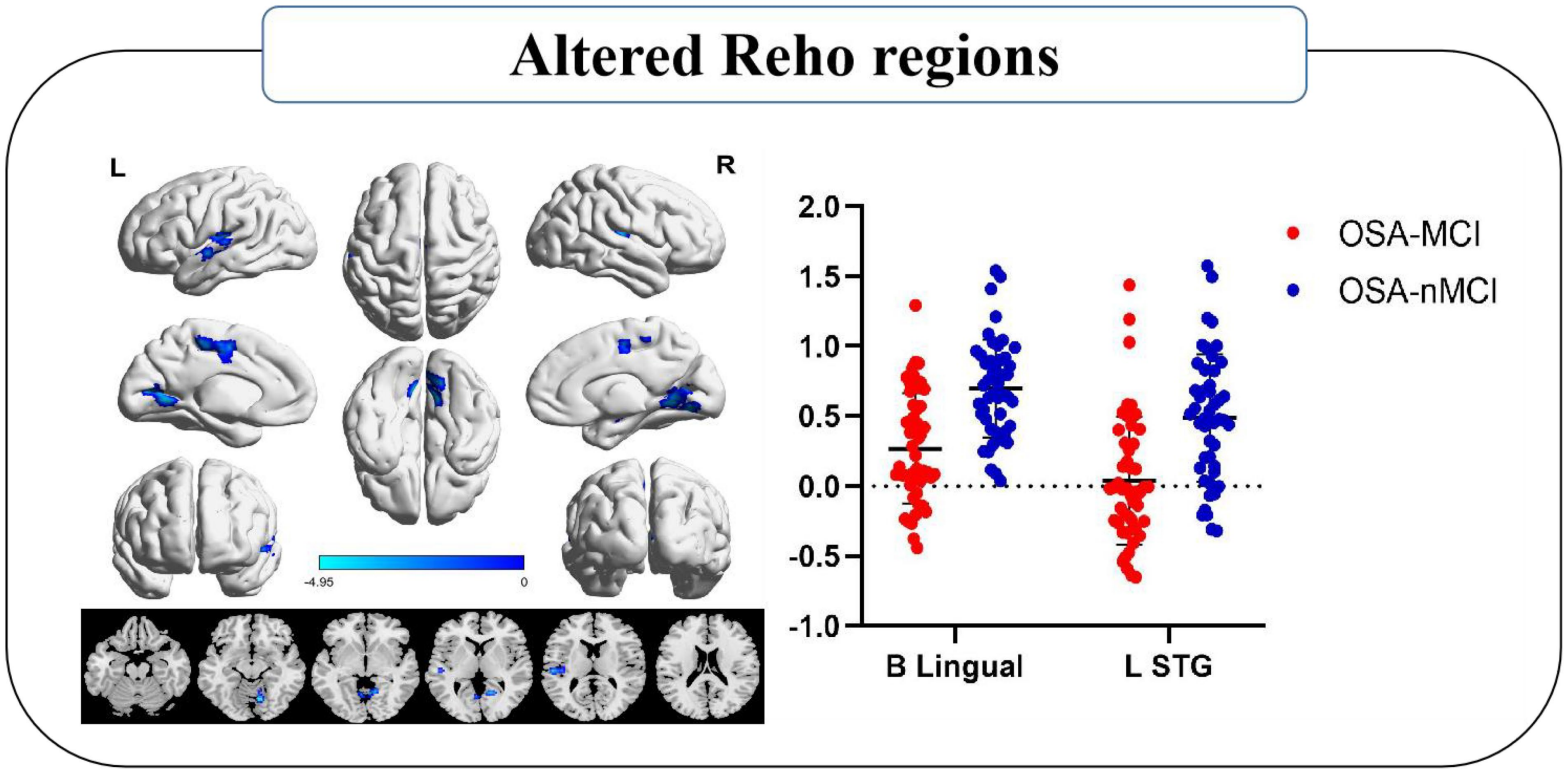
Significantly different Reho was observed between the OSA-MCI and OSA-nMCI groups. Reho values decreased significantly in the MCI group compared to the nMCI group (blue). B, bilateral; L, left; STG, superior temporal gyrus.

The group means of the Reho plots for OSA-MCI and OSA-nMCI are shown in Figure 1. Two-sample *t*-test was performed to compare Reho data between OSA-MCI group and OSA-nMCI group. Differences between groups are shown in Table 2 and Figure 2. Compared with OSA-nMCI, OSA-MCI group showed significant abnormalities in bilateral lingual gyrus and left superior temporal gyrus.

**Table 2:** Altered Reho regions between OSA-MCI and OSA-nMCI groups.

Brain regions	MNI			Voxels	*T* value
	*X*	*Y*	*Z*		
B Lingual Gyrus	12	−69	−12	229	−4.945
L Superior Temporal Gyrus	−54	−9	−3	126	−4.433

MNI, montreal neurological institute; L, left; B, bilateral.

### Group differences in functional connectivity maps

**Figure 3:**
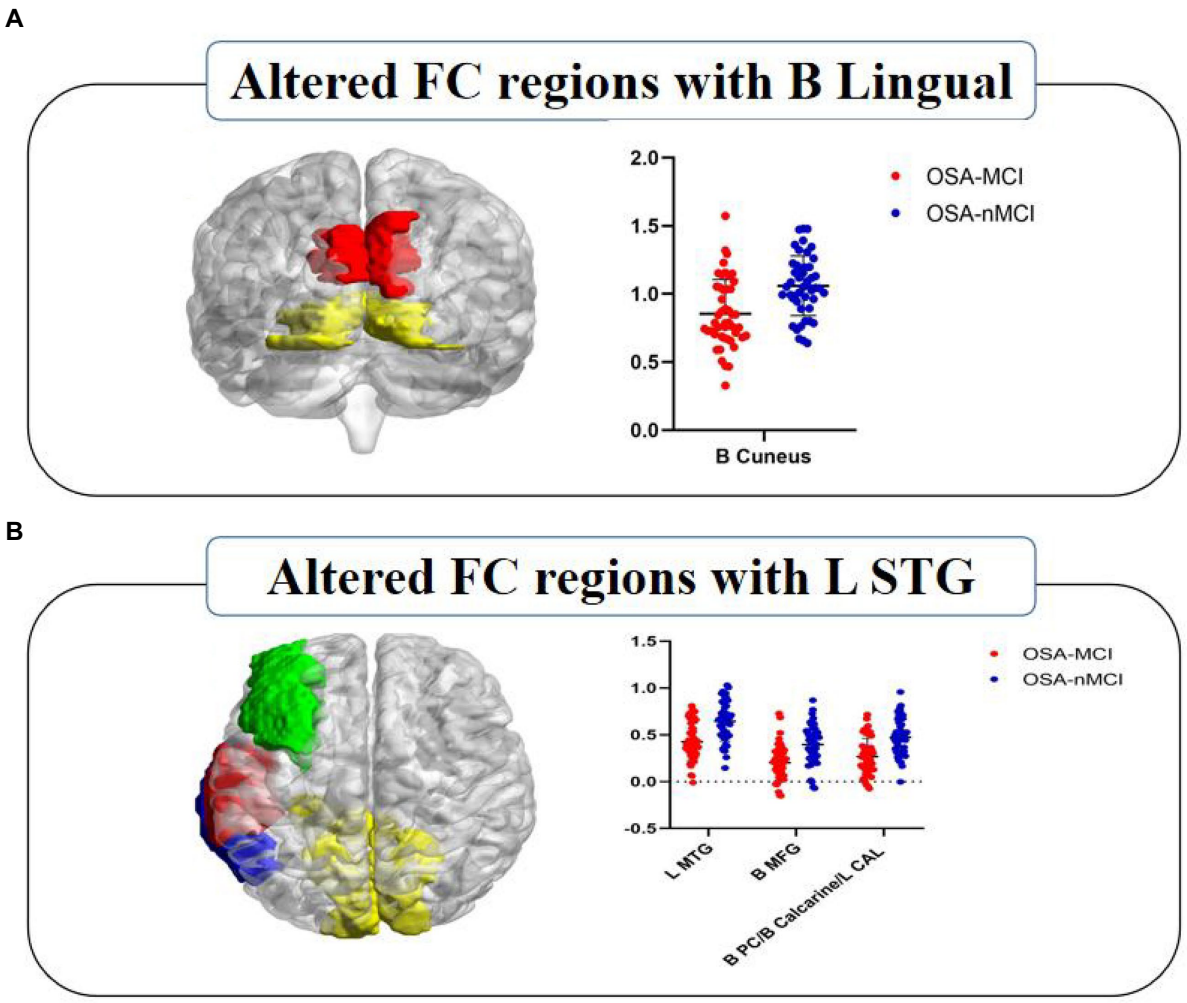
Significantly different FC values seeded as ROI in B-Lingual **(A)** and L-STG **(B)**. In figure **(A)**, red represents the lingual gyrus and yellow represents the cuneus; In figure **(B)**, red represents the left superior temporal gyrus, green represents the left middle frontal gyrus, blue represents the left middle temporal gyrus, and yellow represents the bilateral posterior cingulate/calcarine/left cerebellum anterior lobe. The FCs between significantly different regions and ROIs in OSA-MCI group was significantly reduced compare with OSA-nMCI group. L, left; B, bilateral; ROI, region of interest; FC, functional connectivity; STG, superior temporal gyrus; MTG, middle temporal gyrus; MFG, middle frontal gyrus; PC, posterior cingutate; CAL, cerebellum anterior lobe.

Patients with OSA-MCI showed decreased FC between left superior temporal gyrus (STG) and left middle temporal gyrus (MTG), left middle frontal gyrus (MFG), bilateral posterior cingutate (PC) /Calcarine/left cerebellum anterior lobe (CAL), and decreased FC between bilateral lingual gyrus and bilateral Cuneus (Table 3; Figure 3).

**Table 3:** Functional connectivity between ROIs and the whole brain.

ROIs	Brain regions	MNI coordinates			Voxels	Value of *p*
		*X*	*Y*	*Z*		
L STG	L MTG	−66	−30	−9	209	−5.101
	L MFG	−6	39	−12	341	−5.067
	B PC/B Calcarine/L CAL	−21	−36	−21	1,044	−5.479
B Lingual	B Cuneus	6	−81	33	254	−4.500

ROIs, regions of interest; MNI, montreal neurological institute; L, left; B, bilateral; STG, superior temporal gyrus; MTG, middle temporal gyrus; MFG, middle frontal gyrus; PC, posterior cingutate; CAL, cerebellum anterior lobe.

### Correlational analysis

**Figure 4:**
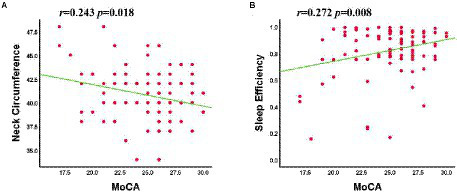
The MoCA scale scores was negatively correlated with neck circumference **(A)** and positively correlated with sleep efficiency **(B)**.

In patients with OSA, the MoCA scale scores showed a significant positive correlation with sleep efficiency (*r* = 0.272, *p* = 0.008) and a negative correlation with neck circumference (*r* = −0.243, *p* = 0.018; Figure 4). No signifificant correlation was found between clinical variables, MoCA scale scores and the abnormal Reho and FC value in OSA-MCI or OSA-nMCI groups.

### Logistic regression

**Figure 5:**
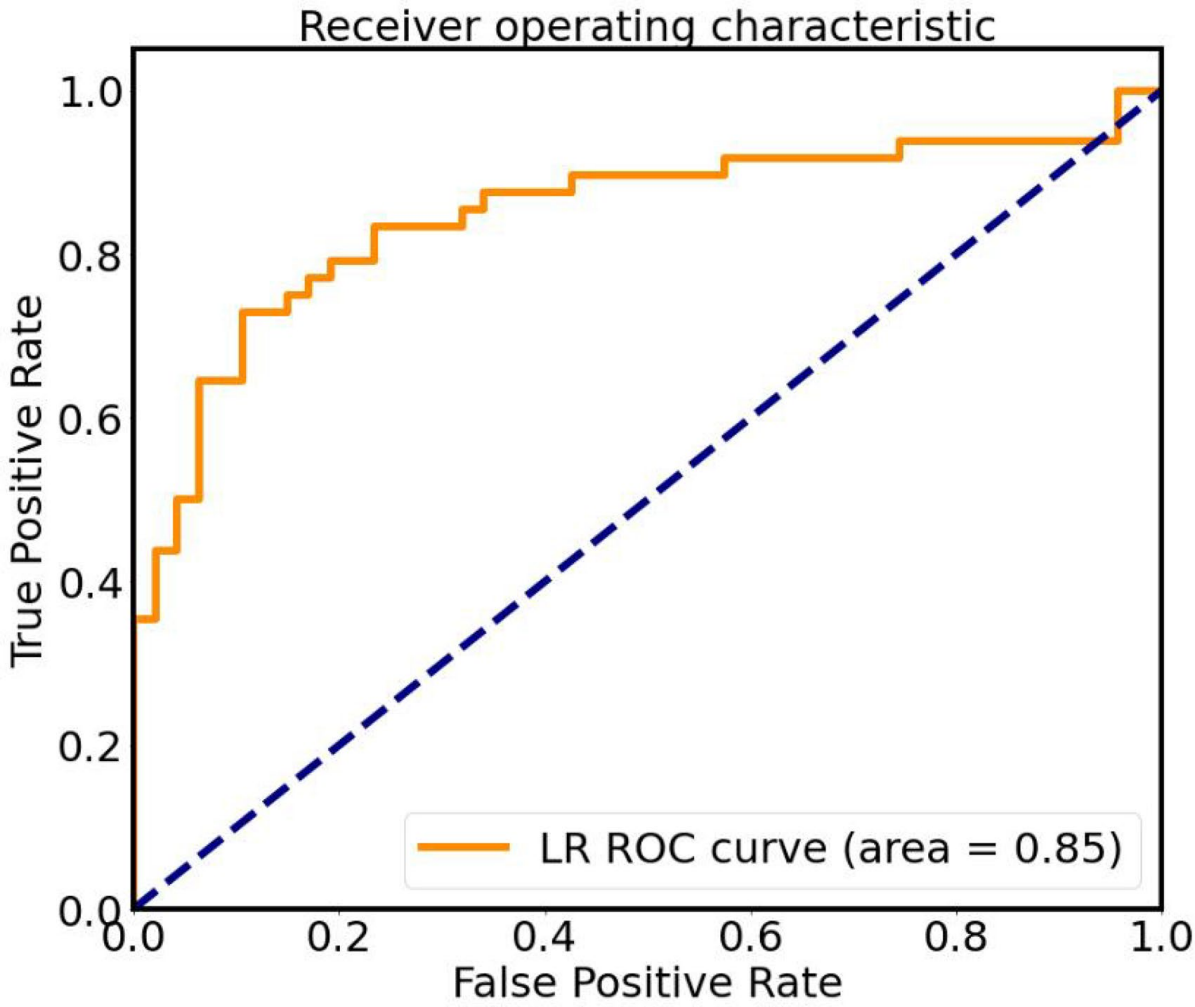
ROC curve of classifier based on altered Reho and FC values, AUC is 0.85. LR, logistic regression; ROC, receiver operating characteristic.

The screened features (Reho of bilateral lingual gyrus, left STG, FC between left STG and B PC/B Calcarine/L CAL) were optimized with hyperparameters and left cross validation to obtain the performance of LG machine learning model, as shown in Figure 4. The AUC was 0.85, the accuracy was 0.87, the sensitivity was 0.70, the specificity was 0.89, the Kappa coefficient was 0.60, and the *p*-value was 0.0014 after 5,000 permutation tests (Figure 5).

## Discussion

In this study, voxel-based Reho and secondary FC were used to explore the abnormal brain activity in OSA-MCI and OSA-nMCI groups. Consistent with our hypothesis, compared with OSA-nMCI group, OSA-MCI group showed significantly lower Reho value in the bilateral lingual gyri and left STG. Decreased FC value was observed between left STG and MTG, left MFG, bilateral PC, calcarine cortex, left ACL, and between bilateral lingual gyrus and bilateral cuneus,which DMN, visual network (VN), and cerebellar networks were mainly involved. These findings indicating that the functional changes of such brain networks may be related to mild cognitive impairment. Secondly, based on the Reho and FC characteristics of significantly different brain regions, we can effectively distinguish whether OSA patients are accompanied by MCI, and provide more evidence for early clinical intervention.

Our study found that in OSA-MCI group, the Reho value of left STG was decreased, the FC value between left STG and left MTG, MFG, and bilateral PC was decreased, and these abnormal brain functional regions were mainly involved in DMN. The DMN is a group of brain regions, including the anterior and medial frontal cortex, bilateral temporal lobes, precuneus, and lateral parietal cortex, that are associated with social behavior, emotional control, memory, learning, and task execution (Raichle, 2015). Ya-ting Chang et al. showed that reduced functional connectivity in the DMN was associated with nocturnal hypoxemia rather than sleep rhythms (Chang et al., 2020). Our previous studies have shown that decreased FC between the DMN and the central executive network and decreased functional connectivity between brain networks are associated with delayed memory (Li et al., 2016). DMN was further divided into aDMN and pDMN (Yang et al., 2017; Wang et al., 2018), aDMN is related to social cognitive functions (emotional self-referential processing and inferential mental states of others), and pDMN is related to cognitive processes (temporal episodic memory and thinking). Zhang et al. (2013) found that FC in the anterior DMN of OSA patients decreased, while FC in the posterior DMN increased compensatively. Similar to previous studies, we found changes in FC values in multiple brain functional areas in the MCI group, which may be related to long-term intermittent hypoxia in OSA patients. In addition, compared with the nMCI group, the FC changes in multiple brain regions (aDMN and pDMN) were decreased, indicating that MCI patients may have more obvious brain function changes, and the possible disruption of compensatory mechanism in the DMN may be the potential cause of MCI.

In our study, we found that the Reho value of bilateral lingual gyrus and FC of left STG and calcarine were decreased in MCI group. The lingual gyrus is mainly involved in the processing of visual memory (Menon and Uddin, 2010) and is an important region of visual perception (Yang et al., 2015). The calcarine is a component of the primary visual cortex and is associated with spatial working memory response time (Valenzuela et al., 2015). Both lingual gyrus and calcarine cortex are involved in the visual network, and the functional changes of visual network are related to MCI (Amaefule et al., 2021). In patients with Alzheimer’s disease, task-state and resting-state MRI studies found that decreased FC of visual network function was significantly associated with cognitive impairment, and changes in visual network integrity were associated with the progression of MCI (Huang et al., 2021). Yang et al. (2022) showed that compared with healthy people, OSA patients took longer to process visual information, which reflected impaired visual perception from the perspective of electrophysiology, and suggested that impaired visual perception may be the potential mechanism of cognitive impairment. In our study, compared with the OSA-nMCI group, the function of brain areas related to visual network was impaired in the MCI group, which may be related to hypoxemia and oxidative stress (Archer and Pepin, 2013). However, reduced Reho in the bilateral lingual gyrus and FC in the left STG and talus were not associated with cognitive scales. Therefore, the potential causal relationship between changes in the visual network and cognitive dysfunction needs further investigation.

The cerebellum is a key module not only in the motor control system, but also in cognitive and emotional processing (Stoodley et al., 2012). Ping Xiao’s study showed that cerebellar gray matter thinning and decreased blood flow in OSA patients and found that structural changes in cerebellum and changes in blood circulation were associated with cognitive impairment (Xiao et al., 2022). Several other studies have shown that the cerebellum is vulnerable to hypoxia and ischemia, and that sleep deprivation is also an important factor affecting cerebellar function (Gazes et al., 2012; Kim et al., 2016). In a study of healthy people, small-world properties of the cerebellum and brain-cerebellum functional coupling were shown (Chen et al., 2022). A resting-state function-based study found that intermittent hypoxia resulted in impaired cerebellar network integration and cerebellar functional connectivity and was associated with cognitive impairment (Park et al., 2022). In this study, our results showed that FC decreased in the anterior cerebellar lobe and left STG, and this decreased functional connectivity in the cerebellum may be related to hypoxia or ischemia. Therefore, we hypothesized that hypoxia or sleep disturbance may lead to the disruption of functional connectivity between cerebellum and brain, which may be one of the causes of cognitive impairment.

In addition, we found that MoCA scale scores were associated with neck circumference and sleep efficiency. Increased neck circumference is associated with obesity, which is one of the risk factors for OSA. Obesity plays an important role in the pathogenesis of apnea syndrome by altering the caliber of the upper airway during breathing in subjects and thus in the pathogenesis of apnea syndrome (Kim et al., 2014). An arterial MRI study showed that the posterior palatal airway was narrower in obese subjects during respiratory arousal and was associated with OSA severity (Feng et al., 2018). Our results showed that the range of neck circumference was significantly different between the OSA-MCI and OSA-nMCI groups. Therefore, we hypothesized that the increase of neck circumference may imply changes in airway anatomy, which may be related to the severity of OSA, although no significance difference was found in BMI or AHI indices between the two groups. Characteristics of sleep disorders include decreased sleep duration and quality, reduced sleep efficiency, fragmented sleeping and sleepiness all day (Casagrande et al., 2022). Previous studies have also shown that sleep disorders are associated with declined cognitive level (Bubu et al., 2020), particularly impairements showed in attention, memory and executive. Our study showed a positive correlation between sleep efficiency and cognitive performance, suggesting that the decline in sleep efficiency may be associated with cognitive impairment.

At last, based on Reho and FC features, LR was used to distinguish OSA-MCIs and OSA-nMCIs. Khatri and Kwon (2022) showed that an efficient classification model was established based on various rs-fMRI and structural MRI, combined with machine learning, indicating that rs-fMRI can be used as a neuroimaging marker to solve prediction and classification. In this study, LR models with excellent classification accuracy (AUC = 0.85, accuracy rate 0.87, sensitivity 0.70, specificity 0.89) were constructed, which suggests that LR may be an effective tool to identify OSA-MCI from OSA patients.

### Limitation

First, our subjects were male with severe OSA, and the results may not be generalizable to patients with mild OSA or women with OSA. Second, we only analyzed abnormal functional change and ignore potential morphological and microstructural changes, which may help us to deepen our understanding of OSA with cognitive impairment. Therefore in the future, we will combine other fMRI features (e.g., cortical thickness, white matter fiber) to improve the classification efficiency of the model. Finally, the sample size was small. Larger samples will be needed in future studies.

## Conclusion

Our results show that OSA patients with MCI have spontaneous brain activity changes and decreased functional connectivity in multiple brain regions, mainly related to DMN, VN, and the cerebellar network. These provide additional information about the underlying neural mechanisms of OSA-related cognitive impairment. At the same time, our results demonstrate that an effective machine learning method can efficiently distinguish whether OSA patients are accompanied by MCI and provide potential imaging markers for clinical treatment.

## Data availability statement

The raw data supporting the conclusions of this article will be made available by the authors, without undue reservation.

## Ethics statement

The studies involving human participants were reviewed and approved by the Medical Ethics Committee of the First Affiliated Hospital of Nanchang University. The patients/participants provided their written informed consent to participate in this study.

## Author contributions

YS and XL wrote, reviewed, and revised the manuscript. DP guided and designed the MRI experiment. HL analyzed the resting-state fMRI data. YS and HL analyzed and discussed the ideas of the paper. PY analyzed machine learning. WD, KL, YZ, and WX collected resting-state fMRI data and applied for the ethics approval. All authors contributed to the article and approved the submitted version.

## Funding

This study was supported by the National Natural Science Foundation of China (grant nos. 81860307 and 81560285); the Natural Science Foundation Project of Jiangxi, China (grant nos. 20202BABL216036, 20181ACB20023, and 20171BAB205070).

## Conflict of interest

The authors declare that the research was conducted in the absence of any commercial or financial relationships that could be construed as a potential conflict of interest.

## Publisher’s note

All claims expressed in this article are solely those of the authors and do not necessarily represent those of their affiliated organizations, or those of the publisher, the editors and the reviewers. Any product that may be evaluated in this article, or claim that may be made by its manufacturer, is not guaranteed or endorsed by the publisher.

## Footnotes

^1^ www.MRIcro.com
